# Another frontier for harm reduction: contraceptive needs of females who inject drugs in Estonia, a cross-sectional study

**DOI:** 10.1186/s12954-018-0215-0

**Published:** 2018-03-05

**Authors:** Anneli Uusküla, Mait Raag, Sigrid Vorobjov, Don Des Jarlais

**Affiliations:** 1 Department of Family medicine and Public Health, University of Tartu, Ravila 19, 50411 Tartu, Estonia; 2 Infectious Diseases and Drug Monitoring Department, National Institute for Health Development, Tallinn, Estonia; 3 Department of Psychiatry, The Baron Edmond de Rothschild Chemical Dependency Institute, Icahn School of Medicine at Mount Sinai, New York, NY USA

**Keywords:** People who inject drugs, Women, Females who use drugs, Contraception, HIV, Estonia

## Abstract

**Background:**

Despite increasing contraceptive availability, unintended pregnancy remains a global problem. Developing strategies to reverse this trend and increasing occurrence of withdrawal syndrome among newborn children of females currently injecting drugs warrants special attention. The knowledge base on the uptake of effective contraception among females who inject drugs (FWID) is scant. We aimed to examine the prevalence of and factors associated with the use of non-condom contraceptives among sexually active FWID with the focus on effective contraception.

**Methods:**

In a series of cross-sectional studies (2007–2013), 265 current FWID were recruited through respondent-driven sampling (RDS), interviewed, and tested for HIV. RDS weights were used to estimate the prevalence of effective contraception (hormonal contraception, intrauterine device, sterilization) use in the last 6 months.

**Results:**

Of the sexually active women with main partners (*n* = 196) 4.8% (95% CI 2.3–9.7) were using effective contraception, 52.7% (95% CI 42.5–62.7) less-effective or no contraception. 42.5% (95% CI 32.7–52.9) relied on condoms for contraception. The odds for using effective contraception were higher among women with > 10 years of education (OR 7.29, 95% CI 1.4–38.8). None of the women lacking health insurance (*n* = 84) were using effective contraception.

**Conclusions:**

The very low coverage with effective contraception highlights the need to improve contraceptive services for FWID. Reproductive health service including contraception should be considered essential components of harm reduction and of comprehensive prevention and care for HIV among persons who use drugs.

## Introduction

Aside from the immediate health risks of injecting drug use (IDU), such as overdose or transmission of blood-borne infections, IDU often leads to dependence, incarceration, and potentially premature death [1–3]. While a “comprehensive package of interventions” is recommended by the World Health Organization, the United Nations Office on Drugs and Crime, and the Joint United Nations Program on HIV/AIDS, gender-specific needs of women are often ignored or overlooked in the development and delivery of prevention and treatment services [4]. However, the proportion of women among people who inject drugs (PWID) ranges from 10 to 30% and is increasing [5]. Unfortunately, IDU is most prevalent during late adolescence and early to mid-adulthood, coinciding with a woman’s childbearing years [4]. Despite this alignment of risks, only a few studies have assessed the utilization of female-specific contraceptive use among females who inject drugs (FWID) nor have they quantified a higher incidence of pregnancy and abortion of undesired pregnancies [6–9]. Although research exists for condom use among PWID, little is known of non-condom contraception use in women currently injecting drugs, and scarce data are available related to the gender-specific needs for harm reduction services and preferences for contraceptive methods.

The complexities of PWID reproductive life are not limited to the contraceptive challenges.

While the diagnosis of the neonatal abstinence syndrome (NAS) is often dependent on physician bias [10], the rise in NAS incidence has been documented in the USA and Europe [11–13]. A recent assessment indicates that treatment for NAS is often of poor quality across the USA [14]. Further, reviews on effects of opioid agonist treatment for pregnant opioid-dependent women concluded that the evidence base was of very low quality, yet documented an increased risk of NAS among newborns of mothers receiving medication-assisted treatment [15]. Importantly, positive outcomes of the treatment are higher birth weight and parental custody [16]. It has been acknowledged that the opioid epidemic in the USA has brought along an alarming number of children placed into foster care [17].

Eastern Europe and Central Asia have the fastest growing HIV epidemic, initially driven in large part by injection drug use [18], and it is noteworthy that PWID account for 51% of new HIV infections in this region. According to a global review, Estonia has one of the highest concentrations of PWID among those 15–64 years (1.5% in 2007) and a very high HIV prevalence among PWID [1]. Several studies have examined sociodemographic factors and behaviors associated with IDU in Estonia [19–23]. One such study documented that females who inject drugs and who have started injecting recently are in high risk of becoming infected with HIV via sexual transmission, even before IDU initiation, highlighting the need for gender-specific interventions [24]. In this report, we examine the prevalence of and factors associated with the use of non-condom contraceptives among FWID with the focus on effective contraception.

## Methods

### Participant recruitment

The data for the current analysis were collected in a series of four cross-sectional studies conducted in 2007, 2009, 2011, and 2013, in Tallinn, the capital of Estonia. The methods employed in this study, integrated biological and behavioral surveys using standardized recruitment criteria and respondent-driven sampling (RDS) survey methodology, have been described previously [19–23]. RDS is particularly effective in hard-to-reach populations, such as people who inject drugs, because it utilizes a referral system to recruit individuals via networks and compensate for the dependency between study participants [25, 26].

Potential participants were eligible to be included in the study if they were at least 18 years of age, were Estonian or Russian language speakers, reported having injected in the previous 2 months, and were able and willing to provide informed consent. Recruitment began with the non-random selection of “seeds” (*n* = 5, 2007; *n* = 8, 2009; *n* = 6 in 2011 and 2013) purposefully selected (among PWID known to field team) to represent diverse PWID types (by age, gender, ethnicity, main type of drug used, and HIV status). After they had participated in the study, the subjects were provided with coupons for recruiting up to three of their peers (other persons who inject drugs). The coupons were uniquely coded to link participants to their survey responses and to biological specimens, and for monitoring who recruited whom. Participants who completed the study received a primary incentive (a grocery store voucher, 10€ value) for participation in the study and a secondary incentive (a grocery store voucher, 5€ value) for each peer successfully recruited (i.e., came to the study site, was deemed eligible, and completed study procedures).

Study participation was anonymous in 2007–2011 and non-anonymous (but confidential) in 2013.

### Measures

Information on demographic and social factors, injection, and sexual risk behaviors were recorded by trained fieldworkers in a structured confidential interviewer-administered questionnaire using standardized study items and questions adapted from the WHO Drug Injecting Study Phase II survey v2b [27]. For HIV serostatus, venous blood was collected and tested for the presence of HIV antibodies using commercially available test kits (HIV-1/HIV-2 III Plus from Abbott Laboratories (Abbott Park, IL, USA) in 2007, 2009, and ADVIA Centaur CHIV Ag/Ab Combo (SIEMENS) in 2011, 2013).

The subset of female subjects in the 2007, 2009, 2011, and 2013 surveys was used in this analysis. Contraceptive method data was drawn from responses to the survey item “what methods of contraception have you and your main partner used in the last 6 months?” We used the following definition of the main partner: someone who is your most important regular sex partner of the opposite sex. The primary outcome for the study, contraceptive method reported by female subjects, was dichotomized as follows: (1) highly effective contraception (rate of unintended pregnancies < 10 per 100 women: hormonal contraception/intrauterine device/sterilization) [28] and (2) less-effective contraception (rate of unintended pregnancies ≥ 10 per 100 women: including condom, diaphragm/cap/sponge/spermicide/rhythm method/using no contraception [28]).

The interview elicited information on injection behavior (age at injection initiation, injecting frequency, drug of choice), sexual behavior (number of sexual partners in last 12 months; having been paid for sex (ever), sexual partner types (main only, casual only, main and casual), condom use with the main and casual partner(s) (never, sometimes, always) in the last 6 months), self-reported health, contextual (imprisonment experience, health insurance coverage) and sociodemographic factors (age, ethnicity, education, marital status, main source of income), self-reported HIV status, and receiving HIV treatment.

Study participation in 2007, 2009, and 2011 was anonymous and in all years with the protocol including pre- and post-HIV test counseling for participants.

Ethical approvals were obtained from the Tallinn Medical Research Ethics Committee (2007) and Ethics Review Board of the University of Tartu (2009–2013) and the Beth Israel Medical Center Institutional Review Board for 2007–2013 in New York, USA. Written informed consent was secured from all participants.

### Study sample

To avoid potential duplication of individual subjects in the analyses (in samples recruited in 2007–2011), a set of selected personal characteristics (ethnicity, month of birth, year of birth, country of birth, and age at first injection drug use) was used to identify potential duplicate study participants across years of study implementation. No duplicates were identified among female study participants.

Of the total of 1353 current PWID recruited to the four studies, 19.5% (*n* = 265) were women.

### Statistical analysis

Recruiting chains of the four individual RDS studies were joined to form one RDS dataset, preserving the original recruitment chains. The structure of the joint RDS data was similar to that of an individual RDS study, the number of seeds equaling the sum of the number of the seeds of the four studies. Data on egocentric network sizes (the number of potential participants that the respondent knew within the target population) and recruitment chains were used to derive RDS sequential sampling weights [29] with the package RDS [30] within the R statistical environment v 3.2.4 [31]. Using the survey package [32], weighted means and proportions with 95% confidence intervals (CI) were used to summarize continuous and categorical variables, respectively. Taking into account the RDS weights, continuous variables were compared by the Kruskal-Wallis test, categorical variables by the chi-square test (with Rao-Scott correction) [33]. RDS-weighted logistic regression was used to compute odds ratios (OR) with 95% CIs.

Sample proportions and RDS-weighted estimates are presented in Table 1. Following RDS weighting, the population estimates were generally similar to the unweighted values. All observed sample proportions fell within the 95% confidence intervals of the RDS-adjusted population estimates.

**Table 1:** Effective contraception use among women injecting drugs (sample proportions and RDS proportions together with 95% confidence intervals) in Tallinn, Estonia (in 2007–2013)

	Reporting the use of effective contraception			
	All women *N* = 265		Sexually active FWID with main sexual partner(s) *N* = 196	
	Sample proportions *n*/*N* (%)	RDS proportions (95% CI)	RDS proportions (95% CI)	*p* value
Sociodemographic characteristics				
Age				0.852
< = 30	6/190 (3.2%)	4.4% (1.7–11.0)	4.6% (1.8–11.3)	
> 30	4/74 (5.4%)	5.1% (1.6–14.7)	5.2% (1.7–15.3)	
Ethnicity				0.188
Estonian	2/32 (6.3%)	10.9% (2.3–38.6)	10.9% (2.3–39.3)	
Non-Estonian	8/233 (3.4%)	3.6% (1.6–8.2)	3.7% (1.6–8.5)	
Education				0.007
< 10 years	2/139 (1.4%)	1.1% (0.3–4.5)	1.1% (0.3–4.6)	
10+ years	8/124 (6.5%)	7.4% (3.2–16.0)	7.7% (3.3–16.6)	
Main income				0.591
Employment	2/70 (2.9%)	2.8% (0.6–11.6)	3.0% (0.7–12.3)	
Subsistence	5/90 (5.6%)	7.0% (2.4–18.7)	7.0% (2.4–18.8)	
Other	3/105 (2.9%)	3.8% (0.9–14.6)	4.0% (0.9–15.1)	
Sex work (ever)				0.229
No	8/216 (3.7%)	5.4% (2.4–11.7)	5.6% (2.5–12.0)	
Yes	2/49 (4.1%)	2.1% (0.5–8.8)	2.1% (0.5–9.1)	
Incarceration (ever)				0.442
No	6/149 (4.0%)	5.5% (2.2–13.0)	5.6% (2.2–13.4)	
Yes	4/116 (3.4%)	3.2% (1.0–9.3)	3.2% (1.1–9.6)	
Marital status				0.073
Divorced	2/24 (8.3%)	22.9% (5.0–62.8)	27.1% (5.1–72.3)	
Married	8/134 (6.0%)	5.4% (2.3–12.1)	5.5% (2.4–12.4)	
Single	0/94 (0%)	0%	0%	
Widow	0/12 (0%)	0%	0%	
Sexual behavior				
Number of sexual partners (last 12 months)				0.553
0	0/27 (0%)	0%	na	
1	7/142 (4.9%)	4.3% (1.7–10.6)	4.5% (1.8–10.9)	
2	2/43 (4.7%)	9.3% (2.0–33.6)	9.3% (2.0–33.9)	
3+	1/52 (1.9%)	2.2% (0.3–14.9)	2.3% (0.3–15.6)	
Type of sexual partners (last 6 months)				0.025
Casual only	0/18 (0%)	0%	na	
Main and casual	0/22 (0%)	0%	0%	
Main only	10/178 (5.6%)	5.5% (2.6–11.2)	5.7% (2.7–11.5)	
Condom use with casual partners (last 6 months)				
Never	0/10 (0%)	0%	0%	
Sometimes	0/7 (0%)	0%	0%	
Always	0/24 (0%)	0%	0%	
Injection drug use				
Age at first injecting				0.309
< = 20	0/36 (0%)	0%	0%	
> 20	10/228 (4.4%)	5.4% (2.6–10.9)	5.5% (2.6–11.2)	
Daily no. of injections				0.392
1	4/86 (4.7%)	6.3% (2.1–17.8)	6.4% (2.1–18.1)	
> 1	6/177 (3.4%)	3.4% (1.4–8.1)	3.5% (1.4–8.4)	
Injecting frequency (last 4 weeks)				0.232
Less than daily	6/159 (3.8%)	4.1% (1.6–10.2)	4.1% (1.6–10.4)	
Daily	2/101 (2.0%)	1.4% (0.3–6.0)	1.5% (0.3–6.4)	
Main drug				0.267
Fentanyl	5/168 (3.0%)	2.2% (0.8–5.8)	2.2% (0.8–5.9)	
Other	3/91 (3.3%)	5.1% (1.4–16.8)	5.3% (1.5–17.5)	
Health and HIV				
Health insurance				0.111
No	0/84 (0%)	0%	0%	
Yes	9/177 (5.1%)	6.0% (2.7–12.9)	6.2% (2.8–13.1)	
HIV				0.122
Neg	5/109 (4.6%)	7.1% (2.7–17.5)	7.3% (2.7–17.9)	
Pos	5/156 (3.2%)	2.5% (0.9–6.6)	2.6% (1.0–6.8)	
Aware of HIV+ status				0.306
No	0/35 (0%)	0%	0%	
Yes	5/121 (4.1%)	3.3% (1.2–8.8)	3.4% (1.2–9.0)	
HIV-infected on ART				0.213
No	2/91 (2.2%)	1.3% (0.3–5.4)	1.4% (0.3–5.7)	
Yes	3/55 (5.5%)	4.2% (1.1–14.2)	4.3% (1.2–14.6)	

## Results

There were 265 FWID with a mean age of 28.5 (range 18–50) years. The mean age for the first illicit drug injection was 19.5 years (range 11–40) years, and the mean number of injections in a typical day was 2.

Of the recruited FWID, 85.6% were of non-Estonian ethnicity (Russian speaking) (*n* = 233), 43.6% reported less than 10 years of education, and 37.0% were previously incarcerated. In terms of income, 39.1% reported government/social subsistence, and 23.3% reported employment as their main source of income. Just over half (50.8%) were married, 36.9% single, 10.6% divorced, and 6.6% widowed. In our study, about two thirds (63.0%) of the women who participated were mothers. Of those with a child, the mean number of children was 1.8 (range 1–6); one in four (25.7%) mothers lived with minor child(ren) younger than 18 years. Most (68.6%) women reported Health Insurance Fund coverage and average health; 35.4% reported excellent or good health and 15.0% reported poor or very poor health.

Half of the participating women were HIV-infected (50.1%, 95% CI 39.0–61.2%); of those HIV-infected, 46.9% were on ART. Of note, ART coverage rose dramatically from 19.2% in 2007 to 56.8% in 2013.

For the more detailed analysis of contraception methods and factors associated with choice of method, we focused on sexually active women who reported having a main partner (*n* = 196). Those not meeting these criteria were 28 women who were not sexually active, 35 who were sexually active but did not have a main partner, and 6 who were trying to conceive.

Sexually active women with a main partner who were retained for analysis were more likely to be married than those who were excluded (57.9 vs 10.3%, respectively, *p* < 0.001). They also had more sexual partners during the previous 12 months (mean 2.5 vs 2.0, *p* < 0.001), and a higher proportion with more than three sexual partners (23.5 vs 10.8%, *p* < 0.001). The women with a main sexual partner also tended to report employment as a main source of income more frequently than their counterparts (28.0 vs 10.2%, *p* = 0.054).

### Contraception methods used among sexually active FWID with a main partner

Of the sexually active women with main partners retained for the analysis (*n* = 196) close to half (*n* = 96; 49.5%, 95% CI 39.5–59.7) reported using no contraception. Condoms were the contraceptive method of choice for 78 women (46.7%, 95% CI 36.3–57.5%), whereas few were on hormonal (oral) contraception (*n* = 4; 2.0%, 95% CI 0.6–7.1), had intrauterine device (IUD) (*n* = 7; 4.0%, 95% CI 1.7–9.2), were sterilized (*n* = 3; 2.1%, 95% CI 0.6–7.0), used spermicide (0.2%), or rhythm method (0.2%). None reported using female condoms, diaphragms, or medroxyprogesterone acetate (muscle injection).

### Sexual behavior and partners of sexually active FWID with a main partner

Over a tenth (13.7%) of the women with a main partner reported also having sex with casual partners within the past 6 months. Condom use with the casual partners did not differ from those with and without a main partner (always, 63.2 vs 48.5%; *p* = 0.632). Of the FWID with main partners, one third (30.0%) reported always and over half (55.1%) never using condom with their main partner. Of the main partners of the FWID, the majority (80.7%, 95% CI 70.6–89.7) were also injecting drugs and one third (31.4%, 95% CI 19.5–43.2) were HIV-infected (according to the female respondent).

### Factors associated with highly effective contraception use

Of the 196 sexually active FWID in need of contraception (with a main partner and not wanting to conceive), 4.8% (95% CI 2.3–9.7) were using effective contraception, 52.7% (95% CI 42.5–62.7) less-effective or no contraception, and the rest (42.5% (95% CI 32.7–52.9)) relied on condoms for contraception (Table 1).

The use of effective contraception among our subjects was associated with education: women with over 10 years of schooling were more likely to report effective contraception use than women with less education (OR 7.29, 95% CI 1.4–38.8). There was a tendency for married women (in comparison to those divorced) to have lower odds for using effective contraception (OR 0.16, 95% CI 0.02–1.07, *p* = 0.07). Women without health insurance or unaware of their HIV-positive status did not use effective contraception. None of the women lacking health insurance were using effective contraception (Fisher’s exact test based on crude counts *p* = 0.059).

Given the very low proportion of FWID who reported using effective contraception, and a large number of possible predictor variables, some with no variation in the group using effective contraception, it was apparent that the data were not well suited for multivariable analyses. Thus, we present the bivariate associations for the correlates of effective contraception use.

### Contraception method and condom use by (self-reported) HIV status of women and their main partners

Of the women with a main partner, 40.9% (95% CI 28.3–53.5%) self-reported being HIV-infected. Condoms were always used with the main partner by 30.0% of the FWID. Less than one third of (self-reported) HIV status-concordant main partnership couples always use condoms (with no difference in reported consistent condom use between HIV-infected and uninfected couples: 29.9 vs 23.7%, *p* = 0.7). Consistent condom use (always) was highest (40.7%) in couples where the female partner was HIV-infected and man uninfected and lowest in couples where female partner was HIV-uninfected and man infected (21.5%) (*p* = 0.2). Uninfected women with an HIV-infected main male partner reported no effective contraception use, and 70.1% of them reported never using condoms (Table 2).

**Table 2:** Condom use with the main partner and contraception use by self-reported HIV status of sexually active FWID and their main partners (RDS estimates: proportions and 95% confidence intervals), in Estonia, in 2007–2013

		HIV status: concordant		HIV status: discordant	
	All	F neg/M neg	F pos/M pos	F pos/M neg	F neg/M pos
	*N* = 196	*n* = 80	*n* = 49	*n* = 39	*n* = 27
Condom use with the main sexual partner (last 6 months) (%, 95% CI)^*^					
Never	55.1% (44.7–65.1)	61.2% (44.9–75.4)	53.8% (32.3–74.0)	33.7% (16.9–56.0)	70.1% (43.0–87.9)
Sometimes	14.9% (9.3–23.1)	8.9% (3.9–19.1)	22.5% (10.8–40.8)	25.6% (9.3–53.6)	8.5% (2.3–26.8)
Always	30.0% (20.9–41.0)	29.9% (16.8–47.3)	23.7% (8.0–52.5)	40.7% (20.4–64.8)	21.5% (6.9–50.3)
Contraception used with main sexual partner (last 6 months) (%, 95% CI)^**^					
Highly effective	4.8% (2.3–9.7)	7.2% (2.7–17.8)	2.2% (0.5–8.9)	4.5% (1.2–15.7)	0.0% (0.0–0.0)
Less effective	95.2% (90.3–97.7)	92.8% (82.2–97.3)	97.8% (91.1–99.5)	95.5% (84.3–98.8)	100.0% (100.0–100.0)

*Condom use (never/sometimes/always) vs concordance (concordant/discordant), *p* = 0.5275, condom use vs HIV status of women among HIV concordant couples, *p* = 0.2507; condom use vs HIV status of women among HIV-discordant couples, *p* = 0.1336; **Contraception (effective/non-effective) vs concordance (concordant/discordant), *p* = 0.3555; contraception vs HIV status of women among HIV concordant couples, *p* = 0.2005; contraception vs HIV status of women among HIV discordant couples, *p* = 0.1326

## Discussion

This study of factors associated with contraceptive use among FWID in Tallinn, Estonia, revealed that needed effective contraception (hormonal contraception/intrauterine device/sterilization) use among women (sexually active, with a main sexual partner and not trying to conceive) was exceedingly low—less than 5% benefit from adequate contraception. Half of women (47%) were not using any contraception at all.

A systematic review of contraceptive use among women with substance use disorders also found that these women have an unmet need for contraception [34]. Comparable to our findings, very low (effective) contraception use among current FWID (not recruited from treatment settings) has been reported (Russia [9]; USA [6, 7]); France [35]). Clearly, work remains to reach this group.

Women’s attitudes towards contraception may be affected by several factors, including amenorrhea [36], low knowledge [37], barriers (particularly stigma) related to seeking health/care services, financial constraints, and generally, a challenging life. In our study, education did emerge as an important factor positively associated with effective contraception use. We consider it noteworthy that *none* of the women lacking health insurance were using effective contraception.

Contraceptive behavior among FWID significantly differs from that of the general population. Compared to the Estonian general population, FWID are more than six times less likely to use effective contraception and over three times more likely to not use any method of birth control [38]. However, this trend diverges importantly among FWID in this study in that they *did* report condom use for contraception (46.7% compared to 31% in the Estonian general population). While condoms are the only contraceptive choice that will also prevent HIV transmission, this is largely within male control. The discordant pair condom use rates found in the study are noteworthy (HIV+ men with HIV− female partners had low condom use). Evidently, women in these partnerships are not only placing themselves at risk for undesired pregnancy but also importantly at great risk for HIV.

Research on opioid (the most frequently injected substances) use during pregnancy has documented the negative effects on the pregnant woman, fetus, and neonate [39, 40]. Infants born to women chronically using an opioid are at risk for neonatal abstinence syndrome. In spite of lack of clarity and consistency in how the syndrome is defined, there are clear signs of growing number of newborns diagnosed with this condition in the USA and in Europe [12, 41]. Further, Cornford et al. [42] noted that 64% of children aged < 16 years born to opioid drug-using women in drug treatment did not live with their mother. In our study, about two thirds of the women were mothers but fewer than a third were living together with their child(ren). Taking care of children while living with the stigma of IDU creates another layer of complexity for the FWID and their family members. Clearly, the relationship between child protection issues and contraception remains complex and poorly described [42]. It has been questioned, whether women who use drugs are too readily separated from their children, temporarily or permanently [43]. Whether the potential premature separation of IDU mothers from their children may predispose the mothers to continue IDU and other risky relationships and behaviors warrants more attention.

We concur with other researchers in the assertion that targeted interventions are warranted to meet the specific (highly effective) contraceptive needs of FWID. The challenging lifestyle of FWID may hinder their access or commitment to medical and social services. However, a study conducted in Australia among women who inject drugs found that FWID strive to control their fertility and express individual contraceptive preferences and concerns for their children (both born and unborn). Women’s drug use should not be automatically associated with an inability to make informed health care choices nor a lack of desire to care for their children [44]. Any efforts to decrease unintended pregnancy will need to include elimination of the everyday barriers which preclude access among at-risk women, such as stigma, lack of insurance, or inadequate coverage. Alternative providers and settings should also be considered, such as during antenatal care and after delivery, at prisons upon release, and drug treatment and needle exchange programs. About 40% of FWID in our study have been imprisoned at some point, and previously incarcerated women are at high risk for unintended pregnancy upon release [45]. Finally, while it does not confer any disease transmission protection, emergency contraception is likely to prevent an unintended pregnancy and should be considered as part of a panel of services provided.

Of the limitations inherent to any study, the cross-sectional design used mostly affects and limits our ability to draw causal inferences. The modest sample size (especially for the FWID using effective contraception) increases the likelihood of type II error. While the fact that we found so few FWID who were using effective contraception that we could not do standard multivariable logistic regression is a limitation, this is an important finding in its own regard. Regression analysis attempts to discern between causal and confounding factors; we propose that in this study, there are probably multiple important causal factors, such as lack of health insurance. Many factors may be necessary but not sufficient causes. Last but not the least, our study focused on women in a main partnership, therefore omitting relevant and needed information on women with casual partnerships.

This study benefits from a carefully selected target population, focusing on only those women who are at risk of becoming pregnant and are not trying to conceive, thus at greatest need for effective contraception. Second, we selected the recall period of 6 months for contraception and partnership measures to be able to capture meaningfully current practices and to lower recall bias. Finally, we described each contraceptive method separately and, for further analysis, grouped the methods with regard to effectiveness.

A recent review detailed research gaps on HIV in women who use drugs [46] highlighted potential vulnerability of FWID for HIV, HCV and other co-morbidities, and access to care for HIV treatment and prevention. Our study highlights the exigency of providing FWID with a range of gender-specific sexual and reproductive health services, including contraception and pregnancy services, in addition to prevention of mother-to-child HIV transmission.

With the continued high prevalence of HIV among PWID, promoting condom use among drug users remains important for preventing sexually transmitted infections. However, the extremely low rate of effective contraception use in Estonia specifically, and notably so in HIV-discordant pairs, underscores the urgent need to promote the use of effective contraceptives (such as hormonal contraception or IUD) for females who are not seeking to become pregnant.

## Conclusion

Reproductive health service including contraception should be considered essential components of harm reduction and of comprehensive prevention and care for HIV among persons who use drugs. Programs that aim to provide free, integrated needle exchange, drug treatment and non-stigmatizing reproductive advice, and parenting assistance would greatly improve access to reproductive health services and expand choices available to women.

## Abbreviations

CI: Confidence intervals
FWID: Females who inject drugs
HIV: Human immunodeficiency virus
NAS: Neonatal abstinence syndrome
OR: Odds ratio
PWID: People who inject drugs
RDS: Respondent-driven sampling

## References

[1] Mathers BM, Degenhardt L, Phillips B, Wiessing L, Hickman M, Strathdee SA, Wodak A, Panda S, Tyndall M, Toufik A, Mattick RP; 2007 Reference Group to the UN on HIV and Injecting Drug Use. Global epidemiology of injecting drug use and HIV among people who inject drugs: a systematic review. Lancet 2008;372(9651):1733–1745. 10.1016/S0140-6736(08)61311-2 18817968

[2] Mathers BM, Degenhardt L, Bucello C, Lemon J, Wiessing L, Hickman M (2013). Mortality among people who inject drugs: a systematic review and meta-analysis. Bull World Health Organ.

[3] Nelson PK, Mathers BM, Cowie B, Hagan H, Des Jarlais D, Horyniak D, Degenhardt L (2011). Global epidemiology of hepatitis B and hepatitis C in people who inject drugs: results of systematic reviews. Lancet.

[4] Iversen J, Page K, Madden A, Maher L (2015). HIV, HCV, and health-related harms among women who inject drugs: implications for prevention and treatment. J Acquir Immune Defic Syndr.

[5] United Nations Office on Drugs and Crime. Women who inject drugs and HIV: addressing specific needs. 2014 [cited on Nov 27, 2017] Available at: http://www.unodc.org/documents/hiv-aids/publications/WOMEN_POLICY_BRIEF2014.pdf

[6] Kouzi AC, Des Jarlais DC, Tross S, Abdul-Quader A, Friedman SR (1992). Contraceptive behavior among intravenous drug users at risk for AIDS. Psychol Addict Behav.

[7] Harvey SM, Bird ST, De Rosa CJ, Montgomery SB, Rohrbach LA (2003). Sexual decision making and safer sex behavior among young female injection drug users and female partners of IDUs. J Sex Res.

[8] Weber AE, Tyndall MW, Spittal PM (2003). High pregnancy rates and reproductive health indicators among female injection-drug users in Vancouver, Canada. Eur J Contracept Reprod Health Care.

[9] Abdala N, Kershaw T, Krasnoselskikh TV, Kozlov AP (2011). Contraception use and unplanned pregnancies among injection drug-using women in St Petersburg, Russia. J Fam Plann Reprod Health Care.

[10] Sarkar S, Donn SM (2006). Management of neonatal abstinence syndrome in neonatal intensive care units: a national survey. J Perinatol.

[11] Sanlorenzo LA, Stark AR, Patrick SW. Neonatal abstinence syndrome: an update. Curr Opin Pediatr. 2018;17 10.1097/MOP.0000000000000589 PMC5843557 29346142

[12] Patrick SW, Kaplan HC, Passarella M, Davis MM, Lorch SA (2014). Variation in treatment of neonatal abstinence syndrome in US children’s hospitals, 2004–2011. J Perinatol.

[13] Grossman MR, Berkwitt AK, Osborn RR, Xu Y, Esserman DA, Shapiro ED, Bizzarro MJ (2017). An initiative to improve the quality of care of infants with neonatal abstinence syndrome. Pediatrics.

[14] Wilson D, Shiffman J. Newborns die after being sent home with mothers struggling to kick drug addictions. Reuters, Dec. 2015:7.

[15] Klaman SL, Isaacs K, Leopold A (2017). Treating women who are pregnant and parenting for opioid use disorder and the concurrent care of their infants and children: literature review to support national guidance. J Addict Med.

[16] Berg RC, Winsvold A, Kornør H, Øverland S, Smedslund G, Hammerstrøm KT, Storetvedt K, Johnsen J, Hansen H, Tømmervik K. Effects of opioid agonist treatment for pregnant opioid dependent women [Internet]. Oslo, Norway: Knowledge Centre for the Health Services at The Norwegian Institute of Public Health. 2008 [cited on Jan 19, 2018] Available at: http://www.ncbi.nlm.nih.gov/books/NBK464917/29320133

[17] Quast T, Storch EA, Yampolskaya S (2018). Opioid prescription rates and child removals: evidence from Florida. Health Aff.

[18] United Nations Office on Drugs and Crime (2016). World Drug Report, in United Nations publication, Research and Trend Analysis Branch, Editor.

[19] Uusküla A, Des Jarlais DC, Raag M, Pinkerton SD, Feelemyer J (2015). Combined prevention for persons who inject drugs in the HIV epidemic in a transitional country: the case of Tallinn Estonia. AIDS Care.

[20] Uusküla A, Rajaleid K, Talu A, Abel-Ollo K, Des Jarlais DC (2013). A decline in the prevalence of injecting drug users in Estonia, 2005–2009. Int J Drug Policy.

[21] Vorobjov S, Des Jarlais DC, Abel-Ollo K, Talu A, Rüütel K, Uusküla A (2013). Socio-demographic factors, health risks and harms associated with early initiation of injection among people who inject drugs in Tallinn, Estonia: evidence from cross-sectional surveys. Int J Drug Policy.

[22] Vorobjov S, Uusküla A, Des Jarlais DC, Abel-Ollo K, Talu A, Rüütel K (2012). Multiple routes of drug administration and HIV risk among injecting drug users. J Subst Abus Treat.

[23] Uusküla A, Abel-Ollo K, Markina A, McNutt LA, Heimer R (2012). Condom use and partnership intimacy among drug injectors and their sexual partners in Estonia. Sex Transm Infect.

[24] Uusküla A, Raag M, Marsh K, Talu A, Vorobjov S, Des JD (2017). HIV prevalence and gender differences among new injection-drug-users in Tallinn, Estonia: a persisting problem in a stable high prevalence epidemic. PLoS One.

[25] Johnston LG, Sabin K (2010). Sampling hard-to-reach populations with respondent driven sampling. Methodological innovations online.

[26] Heckathorn DD, Semaan S, Broadhead RS, Hughes JJ (2002). Extensions of respondent-driven sampling: a new approach to the study of injection drug users aged 18–25. AIDS Behav.

[27] Des Jarlais DC, Perlis TE, Stimson GV, Poznyak V (2006). WHO Phase II Drug Injection Collaborative Study Group: using standardized methods for research on HIV and injecting drug use in developing/transitional countries: case study from the WHO Drug Injection Study Phase II. BMC Public Health.

[28] World Health Organization (WHO) Family Planning: A Global Handbook for Providers. CCP and WHO; Baltimore, MD and Geneva: 2011. World Health Organization Department of Reproductive Health and Research (WHO/RHR) and Johns Hopkins Bloomberg School of Public Health [cited on Nov 27, 2017] Available on http://www.who.int/reproductivehealth/publications/family_planning/9780978856304/en/

[29] Gile KJ (2011). Improved inference for respondent-driven sampling data with application to HIV prevalence estimation. J Am Stat Assoc.

[30] Handcock MS, Fellows IE, Gile KJ. RDS: respondent-driven sampling, version 0.7 (2012). Available on http://hpmrg.org,http://CRAN.R-project.org/package=RDS).

[31] R Core Team R: A language and environment for statistical computing. R Foundation for Statistical Computing, Vienna, Austria (2016). Available on http://www.R-project.org/

[32] Lumley T. Analysis of complex survey samples (R Package, version 3.31-2) (2016). Available on http://r-survey.r-forge.r-project.org/survey/

[33] Thomas DR, Rao JNK (1990). Small-sample comparison of level and power for simple goodness-of-fit statistics under cluster sampling. JASA.

[34] Terplan M, Hand DJ, Hutchinson M, Salisbury-Afshar E, Heil SH (2015). Contraceptive use and method choice among women with opioid and other substance use disorders: a systematic review. Prev Med.

[35] Carrieri MP, Rey D, Serraino D (2006). Oral contraception and unprotected sex with occasional partners of women HIV-infected through injection drug use. AIDS Care.

[36] Reddy RG, Aung T, Karavitaki N, Wass JAH (2010). Opioid induced hypogonadism. The BMJ.

[37] Clergue-Duval V, Robin S, Fortias M, Dupuy G, Badin-de-Montjoye B, Vorspan F (2017). Use and knowledge of contraceptive methods by patients in two substance use disorders treatment centers in Paris. Harm Reduction Journal.

[38] Lippus H, Laanpere M, Part K, Ringmets I, Rahu M, Haldre K, Allvee K, Karro H. Estonian women’s health 2014. Tartu 2015 [cited on Nov 27, 2017] Available on https://sisu.ut.ee/sites/default/files/naisteterviseuuring/files/uusestre2014_loppraport.pdf

[39] Wong S, Ordean A, Kahan M (2011). Substance use in pregnancy. J Obstet Gynaecol Can.

[40] McQueen K, Murphy-Oikonen J (2016). Neonatal abstinence syndrome. N Engl J Med.

[41] International neonatal consortium. Second annual neonatal scientific workshop at the EMA (2016). [cited on Nov 27, 2017] Available on http://www.ema.europa.eu/docs/en_GB/document_library/Presentation/2016/10/WC500214710.pdf

[42] Cornford CS, Close HJ, Bray R, Beere D, Mason JM (2015). Contraceptive use and pregnancy outcomes among opioid drug-using women: a retrospective cohort study. Petersen I, ed PLoS One.

[43] Malinowska-Sempruch K (2015). What interventions are needed for women and girls who use drugs? A global perspective. J Acquir Immune Defic Syndr.

[44] Olsen A, Banwell C, Madden A (2014). Contraception, punishment and women who use drugs. BMC Womens Health.

[45] Clarke JG, Hebert MR, Rosengard C, Rose JS, DaSilva KM, Stein MD (2006). Reproductive health care and family planning needs among incarcerated women. Am J Public Health.

[46] El-Bassel N, Strathdee SA (2015). Women who use or inject drugs: an action agenda for women-specific, multilevel and combination HIV prevention and research. Journal of acquired immune deficiency syndromes (1999).

